# Thirteen-Year Outcomes of Keratorefractive Lenticule Extraction for Myopia Up to −10 Dioptres

**DOI:** 10.1155/joph/9935745

**Published:** 2025-08-13

**Authors:** Fei Xia, Zhuoyi Chen, Xiaosong Han, Yanze Yu, Meiyan Li, Jing Zhao, Xingtao Zhou

**Affiliations:** ^1^Eye Institute and Department of Ophthalmology, Eye & ENT Hospital, Fudan University, Shanghai, China; ^2^Key Laboratory of Myopia and Related Eye Diseases, NHC, Key Laboratory of Myopia and Related Eye Diseases, Chinese Academy of Medical Sciences, Shanghai, China; ^3^Shanghai Research Center of Ophthalmology and Optometry, Shanghai, China

## Abstract

**Purpose:** To explore the long-term (13-year) outcomes associated with keratorefractive lenticule extraction (KLEx) among patients with myopia up to −10 dioptres.

**Methods:** This prospective, nonconsecutive case series included 29 patients (29 eyes) who underwent KLEx procedures from May, 2010, through March, 2013, at the Fudan University Eye and ENT Hospital. Analyses performed preoperatively and at the 1-month, 1-year, 5-year, 10-year, and 13-year postoperative time points included measures of uncorrected and corrected distance visual acuity (UDVA and CDVA), objective and manifest refractions, intraocular pressure, axis length, slit-lamp examination, and corneal tomography.

**Results:** All surgeries were performed without any complications. A UDVA of at least 20/25 was achieved in 29 eyes (100%), while 10 eyes (43.4%) exhibited unchanged CDVA, and no lines were lost for any eyes. Additionally, 21 eyes (72%) and 28 eyes (97%) were, respectively, within ±0.5 D and ±1.00 D of the target refraction. The mean refractive regression from 1 month to 13 years after surgery was −0.26 ± 0.41 D. No significant changes in posterior central elevation (PCE) or △PCE were noted at the follow-up time points (all *p* > 0.05). Significant postoperative increases in higher-order aberrations and vertical coma were observed postoperatively (*p* < 0.001), and these remained largely stable over the follow-up period (all *p* > 0.05).

**Conclusion:** These 13-year follow-up results highlight the safety, stability, and predictability of KLEx as an approach to treating myopia up to −10 dioptres.

## 1. Introduction

Keratorefractive lenticule extraction (KLEx) was first reported in 2011 as an approach to vision correction by Shah et al. [1] and Sekundo et al. [2]. KLEx used a femtosecond laser to create and remove a precise disc-shaped corneal lenticule through a small incision (2–4 mm), reshaping the cornea to correct myopia. It could facilitate more rapid visual rehabilitation and better improve corneal structural integrity while limiting dry eye incidence and obviating the risk of flap-related complications that could arise in patients who undergo laser in situ keratomileusis (LASIK) [3]. To date, there have been many peer-reviewed studies that demonstrated the safety, efficacy, stability, and predictability of KLEx approaches with follow-up intervals from 3 months to 3 years [4–9], but long-term follow-up data lacked. In 2010, our team performed the first clinical study of KLEx in China [10]. Over 6 million KLEx procedures have been conducted to date in China. This study was developed with the aim of assessing the safety and efficacy of KLEx in patients with myopia up to −10 dioptres 13 years postoperatively.

## 2. Materials and Methods

### 2.1. Ethics

This study was performed in accordance with the Declaration of Helsinki and received approval from the Ethics Committee of the Eye and ENT Hospital, Fudan University, China, with all patients having provided written informed consent.

### 2.2. Subjects

This prospective study enrolled 29 patients (29 eyes) who underwent KLEx treatment at the Refractive Surgery Center of Fudan University Eye and ENT Hospital (Shanghai, China) from May 27, 2010, through March 22, 2013, with one eye per patient having been selected at random.

To be eligible for inclusion, patients needed to be (1) over 18 years of age, (2) have a corrected distance visual acuity (CDVA) of 20/25 or better, (3) have had stable myopia for a minimum of 2 years preoperatively, (4) have a spherical equivalent (SE) of less than −10.00 D (astigmatism less than −6.00 D), and (5) not wear contact lenses during the last 2 weeks.

Patients were excluded if they had (1) keratoconus or suspected keratoconus, (2) evidence of active ocular inflammation or infection, (3) any history of ocular trauma, (4) uncontrolled glaucoma, or (5) uncontrolled systemic and connective tissue diseases with the potential to impact corneal wound healing.

### 2.3. Surgical Procedure

A single surgeon (XTZ) performed all surgical procedures. A VisuMax femtosecond laser system (Carl Zeiss Meditec AG) was used for KLEx procedures, which were performed as in prior reports [11]. The femtosecond laser repetition rate was 500 kHz, the pulse energy was 130 nJ, and the other parameters were as follows: lenticule diameter 6.0–6.5 mm, cap diameter 7.0–7.5 mm, cap thickness 100–110 μm, and a 2–4 mm side cut at 12:00.

After surgery was complete, all patients were treated four times per day for 1 week with topical 0.5% levofloxacin drops and four times per day for 1 month with 0.1% sodium hyaluronate drops. In addition, patients were treated eight times per day with 0.1% fluorometholone drops, gradually reducing the dosage in 3-day intervals until treatment was discontinued.

### 2.4. Measurements

Study subjects were examined preoperatively and at 1 month, 1 year, 5 years, 10 years, and 13 years postoperatively. All examinations included measurements of both uncorrected distance visual acuity (UDVA) and CDVA, objective and manifest refractions, intraocular pressure, axis length, slit-lamp examination, and corneal tomography.

A Pentacam HR instrument was used for the measurement of wavefront aberrations, central corneal thickness (CCT), anterior and posterior average keratometry (Km), total corneal refractive power (TCRP), posterior central elevation (PCE), and the difference between preoperative and postoperative PCE values (△PCE). TCRP measurements were made 3 mm from the apex, while PCE and Km measurements were obtained directly from the central 4 mm region above the 8 mm reference sphere of best-fit.

Corneal wavefront aberrations were assessed at an analytical diameter of 5 mm. Zernike polynomials were used to assess spherical aberration coefficients ((*Z*_4_^0^)), vertical coma (*Z*_3_^−1^), horizontal coma (*Z*_3_^1^), vertical trefoil ((*Z*_3_^−3^), horizontal trefoil (*Z*_3_^3^), and root mean square (RMS) of total higher-order aberrations (HOAs) up to fourth order.

### 2.5. Statistical Analyses

SPSS 23.0 (SPSS Inc., IL, USA) and GraphPad Prism 8 (https://www.graphpad.com) were used for all statistical analyses. Categorical data are given as frequencies, whereas continuous data are means ± standard deviations. The safety index was the ratio between the CDVA at 13 years postoperatively and the corresponding preoperative CDVA and the efficacy index was the ratio between the postoperative UDVA at 13 years and the preoperative CDVA. The relationship between planned and actual SE was assessed with Pearson correlation coefficient values. One-way repeated measures ANOVAs and Wilcoxon signed-rank tests were used for the comparison of preoperative and postoperative parameters that were normally distributed and skewed, respectively, with *p* < 0.05 being deemed significant.

## 3. Results

In total, this study included 29 eyes across 29 patients (11 male, 18 female). Patients were a mean of 26.22 ± 6.76 years old when they underwent the KLEx procedure. The preoperative characteristics of these patients are presented in Table 1. None of the eyes in this study were impacted by any intraoperative or postoperative complications such as infection, corneal ectasia, or diffuse lamellar keratitis.

### 3.1. Efficacy

**Figure 1:**
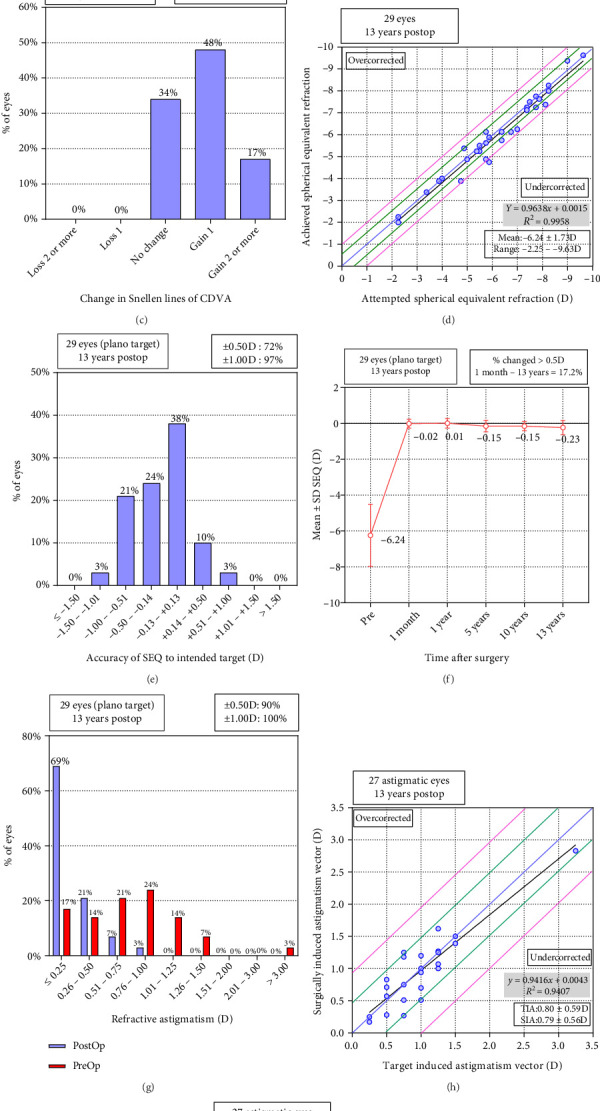
Nine standard graphs of refractive outcomes for 29 eyes at 13 years after KLEx. (a) Cumulative uncorrected distance visual acuity (UDVA); (b) postoperative UDVA versus preoperative corrected distance visual acuity (CDVA); (c) changes in lines of CDVA; (d) spherical equivalent attempted versus achieved; (e)spherical equivalent refractive accuracy; (f) stability of spherical equivalent refraction; (g) refractive astigmatism; (h) target-induced astigmatism versus surgically induced astigmatism; (i) refractive astigmatism angle of error.

The index of efficacy for this study was 1.05 ± 0.17, with a LogMAR UDVA at final follow-up of −0.03 ± 0.08. Of these 29 eyes, 24 (83%) exhibited a postoperative UDVA that was as good as or better than CDVA preoperatively, with 100% of eyes having achieved a UDVA of at least 20/25, 24 (83%) having achieved a UDVA of at least 20/20, and 11 (38%) having achieved a UDVA of at least 20/16 (Figures 1(a), 1(b)).

### 3.2. Safety

The safety index for this study cohort was 1.19 ± 1.18, with a LogMAR UDVA at final follow-up of −0.09 ± 0.07. None of these eyes had lost any CDVA lines, 10 (34%) remained unchanged, 14 (48%) had gained one line, and 5 (17%) had gained two lines (Figure 1(c)).

### 3.3. Predictability

The attempted and actual SE correction achieved in this study is presented in Figure 1(d). Of the 29 eyes, 21 (72%) were within ±0.50 D, while 28 (97%) were within ±1.0 D of the target SE correction (Figure 1(e)).

### 3.4. Stability

The mean manifest SE values at 1 month and 13 years after surgery were −0.006 ± 0.25 D and −0.26 ± 0.41 D, respectively, corresponding to a mean −0.26 ± 0.41 D regression over this 13-year period (−0.02 ± 0.04 D/Year) (Figure 1(f)).

### 3.5. Astigmatism Correction

Of these 29 eyes, 26 (90%) and 29 (100%) were, respectively, within ±0.5 D and ±1.0 D of the targeted astigmatism correction (Figures 1(g), 1(h), 1(i)).

### 3.6. Axial Length

The respective mean axial length measurements prior to and 13 years after surgery were 25.97 ± 0.85 mm and 25.95 ± 0.96 mm, with no significant difference between the two values (*p*=0.122).

### 3.7. Corneal Stability Measurements

#### 3.7.1. CCT, Km, and TCRP

**Figure 2:**
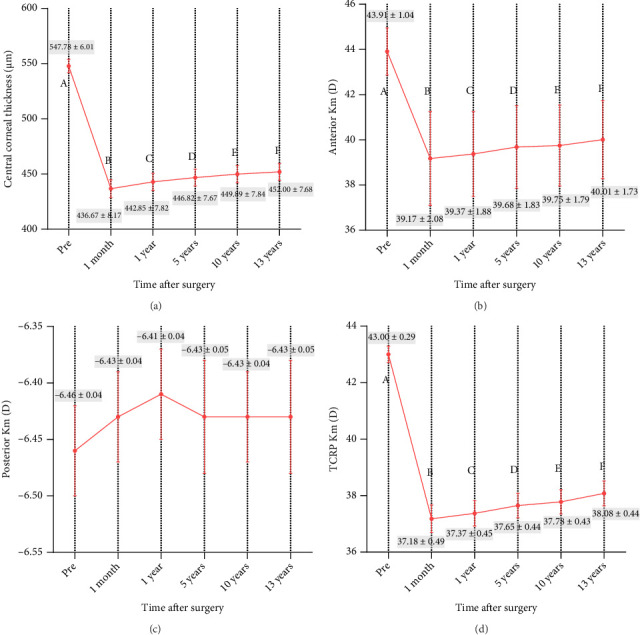
Time course of central corneal thickness, anterior and posterior average keratometry (Km), and total corneal refractive power (TCRP) after KLEx. (a) Central corneal thickness; (b) anterior Km; (c) posterior Km; (d) total corneal refractive power.

At 13 years postoperatively, significant decreases in the mean CCT, anterior Km, and TCRP values were noted relative to their preoperative values (all *p* < 0.001), while no significant differences in posterior Km values were observed over the postoperative follow-up period (*p*=0.095) (Figure 2).

#### 3.7.2. PCE and △PCE

**Figure 3:**
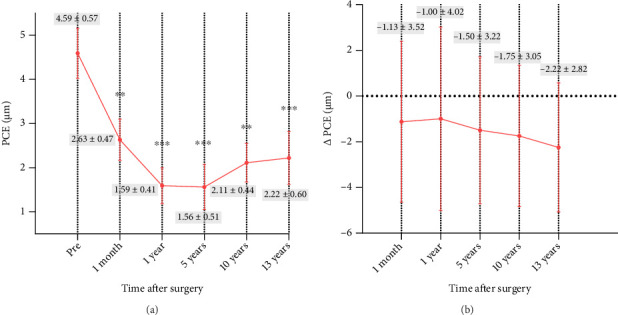
Time course of (a) posterior central elevation (PCE) and (b) the difference between preoperative and postoperative PCE values (△PCE) after KLEx.

**Figure 4:**
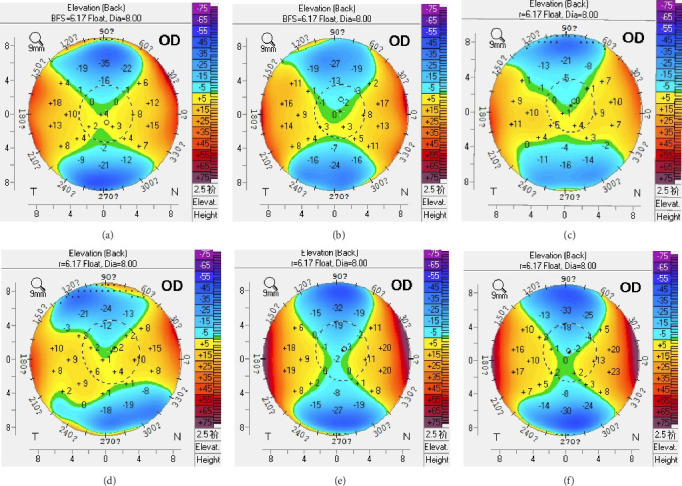
Pentacam posterior corneal elevation map of one participant (right eye): (a) preoperative, (b) 1 month, (c) 1 year, (d) 5 years, (e) 10 years, and (f) 13 years.

At 13 years postoperatively, mean PCE values were significantly lower than preoperative values (*p* < 0.001), with no significant differences in PCE or △PCE values during postoperative follow-up (Figure 3). Changes in posterior corneal surface elevation prior to and following KLEx are presented in Figure 4.

### 3.8. Corneal Wavefront Aberrations

**Figure 5:**
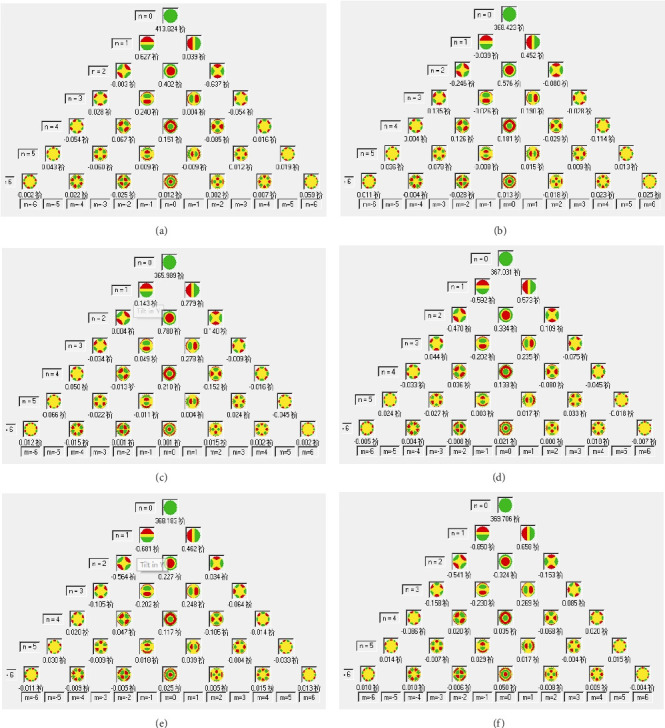
Pentacam corneal wavefront aberration map of one participant (right eye): (a) preoperative, (b) 1 month, (c) 1 year, (d) 5 years, (e) 10 years, and (f) 13 years.

Significant increases in vertical coma and HOAs were evident at the 13-year postoperative time point relative to preoperative analyses (*p* < 0.05). Other aberrations were stable at all follow-up time points (*p* > 0.05). Corneal wavefront aberrations prior to and following KLEx surgery are presented in Table 2.  Figure 5 presents changes in corneal wavefront aberrations prior to and following KLEx.

## 4. Discussion

As our team has pioneered the KLEx technique in China, we have been committed to publishing long-term follow-up research detailing the clinical outcomes of treated patients. We have previously published visual and refractive outcomes associated with the KLEx procedure at 3, 5, 7, and 10 years postoperatively [12–15]. This is the first study, to our knowledge, detailing the ultra-long-term 13-year outcomes for patients with myopia treated via KLEx.

Of the 29 eyes in this study, 24 (83%) achieved a UDVA of at least 20/20%, and 100% exhibited no change or gained at least one line relative to preoperative acuity. None of the analyzed eyes lost any CDVA lines. These results provide support for the long-term safety and efficacy of KLEx as a means of treating myopia. Ten-year follow-up outcome data are the most advanced results to have been published to date pertaining to outcomes among patients with myopia who underwent KLEx [15, 16]. Blum et al. [16] reported that 80% of the treated eyes in their cohort achieved a UDVA of at least 20/25 while 14% had lost one line after 10 years. Xia et al. [15] similarly reported that 72% of treated eyes had achieved a UDVA of at least 20/20 while 6% had lost one line after 10 years. The differences between these prior reports and the present data may be related to the accuracy of preoperative manifest refraction examination measurements taken by optometrists and surgeons or to the optimization of surgical parameters. The patients enrolled in the present study were also relatively young, which may have had some bearing on the study results.

With respect to predictability, 72% and 97% of eyes in this study were respectively within ±0.50D and ±1.0 D of the target SE correction. In their > 5-year follow-up analysis, Papa-Vettorazzi et al. [17] found that 69% and 86% of eyes were, respectively, within ±0.5D and ±1.0D of the target SE correction intended correction during a follow-up period exceeding 5 years, while corresponding percentages of 77% and 95% were reported at 6 years postoperatively by Yildirim et al. [18], in line with the present results. These findings are not universal, however, with some reports having noted predictability values of 48.2% and 59% within ±0.50 D and 78.6% and 81% within ±1.0 D of the target [19, 20]. Predicting long-term outcomes is a challenge for refractive surgery that underscores the need to take many factors into consideration. Our refractive surgery team is among the largest globally, with more than 200,00 cases performed to date. Design efforts for these patients took many long-term predictive factors into account, thus leading to better postoperative patient visual outcomes.

Multiple parameters can impact myopic regression following KLEx, including preoperative SE, preoperative keratometry, age, and various environmental factors [21]. The mean refractive regression observed from 1 month to 13 years postoperatively in the present study was −0.26 ± 0.41 D, consistent with good long-term refractive stability. In our prior study [15], the measured 10-year refraction was −0.32 ± 0.56 D, with this difference in myopic regression being primarily attributable to two additional analyzed eyes with preoperative axial lengths of 26.33 mm and 27.23 mm that had respectively risen to 27.01 mm and 27.42 mm at the 10-year time point. When these two eyes were excluded from that study, the mean regression was −0.22 ± 0.37D (−0.02 ± 0.04 per year). Greater axial length is thus associated with myopic regression. Damgaard et al. [20] and Blum et al. [22] observed respective 7- and 10-year refraction values of −0.25 ± 0.49 D and −0.35 ± 0.66 D, with these values being slightly worse than those in the present study. This may be attributable to the fact that these studies included patients with respective mean ages of 44.2 (range: 31–73) and 46 (range: 31–66) years such that almost half of these patients had reached the presbyopic stage. Mild regression of myopic correction over extended periods may be masked by the normal physiologic hyperopic shift. This study, however, did not include preoperative measurements of axial length. The results of this study have important implications for current and future KLEx treatment. Specifically, patients with stable myopia should be prioritized, whereas for individuals with relatively unstable myopia (axial length), other parameters should be taken into account during the design phase, and detailed communication throughout the entirety of the treatment process is vital to achieve the best possible patient satisfaction.

Some reports focused on other forms of corneal refractive surgery have reported mild long-term regression of myopic correction. Blum et al. [22], for instance, noted a 0.18D 10-year regression for eyes that underwent femtosecond lenticule extraction (FLEx). In 18-year follow-up data reported by Shalchi et al. [23], patients who were under and over 40 years of age at the time of PRK surgery, respectively, exhibited mean SE changes of 0.54 D and 0.05 D. These age-related differences may be related to the fact that younger age tends to be associated with greater axial length, chronic stromal remodeling and wound healing, or the biomechanical instability of the cornea. After 6–7 years, a mean regression of 0.63–0.97 has also been noted for LASIK-treated eyes [24–26].

Posterior elevation is a crucial metric to take into consideration when assessing corneal stability after refractive surgical procedures. None of the eyes in the present study were affected by ectasia, with a slight backward shift in PCE values over the course of the 13-year follow-up period. At different time points, stable △PCE values were observed. Zhao et al. [27] reported preoperative and 3-year postoperative mean PCE values of 0.97 ± 2.29 μm and −1.42 ± 3.48 μm, respectively, for patients undergoing KLEx treatment, in line with the backward shift observed in our study. Chen et al. [28] noted a similar decrease in PCE over a 7-year follow-up interval. These decreases over time may be related to morphological changes stemming from differences in the wound healing processes engaged in the peripheral and central cornea [29, 30]. In this study, PCE was the only analyzed metric that differed. Other posterior corneal elevation changes along different semimeridians on concentric circles were similar and presented with satisfactory stability.

HOAs induced by refractive surgery are related to visual complaints including glare, halos, impaired night vision, and monocular diplopia. In this study, all follow-up analyses revealed increased vertical coma and HOAs in treated patients, with other aberrations having remained stable over the course of follow-up (*p* < 0.05). In line with these results and similar findings published by Tülü et al. [31], many factors are known to influence wavefront aberrations following corneal refractive surgery, including the wound healing process, corneal haze, and the amount of time that has passed since the surgical procedure [32–34]. Surgical incisions for eyes in the present study were generally positioned vertically above the cornea. Intraoperative corneal manipulation can alter the shape of the cornea, contributing to postoperative vertical coma increases.

This study is limited in part by its small sample size. In addition, indices of visual quality such as intraocular scattering of contrast sensitivity were not assessed. This approach was also not compared to other forms of refractive surgery.

Based on these 13-year follow-up results, KLEx is a safe, stable, and predictable approach to treating myopia.

## Figures and Tables

**Table 1:** Preoperative characteristics.

Characteristic	Value
Male/female sex (*n*)	11/18
Age (year)	26.22 ± 6.76 (18–42)
IOP (mm Hg)	11.7 ± 2.22 (7.3–17.1)
Axial length (mm)	25.97 ± 0.95 (23.86–28.42)
Sphere (D)	−5.89 ± 1.66 (−9.0–−2.25)
Cylinder (D)	−0.90 ± 0.61 (0–−3.25)
Spherical equivalent (D)	−6.34 ± 1.79 (−9.63–−2.25)
Ablation depth (μm)	126.24 ± 47.58 (61–317)

*Note:* IOP, intraocular pressure; D, dioptres.

**Table 2:** Preoperative versus postoperative corneal wavefront aberrations for a 5 mm pupil.

Aberrations(μm)	Preoperative	1 month	1 year	5 years	10 years	13 years	*p*
Horizontal coma	0.03 ± 0.07	0.07 ± 0.21	0.07 ± 0.21	0.03 ± 0.23	0.02 ± 0.25	0.01 ± 0.28	*p*=0.231
Vertical coma	−0.01 ± 0.11	−0.27 ± 0.32^a^	−0.33 ± 0.33^a^	−0.28 ± 0.29^a^	−0.28 ± 0.27^a^	−0.33 ± 0.43^a^	*p*=0.002
Horizontal trefoil	0.01 ± 0.05	0.03 ± 0.11	0.04 ± 0.05	0.02 ± 0.08	0.01 ± 0.05	0.01 ± 0.06	*p*=0.60
Vertical trefoil	0.03 ± 0.07	0.03 ± 0.10	0.03 ± 0.10	−0.01 ± 0.06	−0.01 ± 0.07	−0.03 ± 0.08	*p*=0.061
Spherical aberration	0.12 ± 0.04	0.11 ± 0.08	0.10 ± 0.06	0.09 ± 0.06	0.09 ± 0.07	0.08 ± 0.08	*p*=0.496
HOA (RMS)	0.22 ± 0.07	0.44 ± 0.14^a^	0.44 ± 0.16^a^	0.43 ± 0.17^a^	0.42 ± 0.18^a^	0.43 ± 0.21^a^	< 0.001

*Note:* Data are presented as mean ± SD.

Abbreviations: HOA, higher-order aberration; RMS, root mean square.

^a^Versus pre values of statistical significance (*p* < 0.05).

## Data Availability

Data analyzed during the current study are available from the corresponding author upon reasonable request.

## References

[1] Shah R., Shah S., Sengupta S. (2011). Results of Small Incision Lenticule Ex Traction: All-In-One Femtosecond Laser Refractive Surgery. *Journal of Cataract & Refractive Surgery*.

[2] Sekundo W., Kunert K. S., Blum M. (2011). Small Incision Corneal Refractive Surgery Using the Small Incision Lenticule Extraction (SMILE) Procedure for the Correction of Myopia and Myopic Astigmatism: Results of a 6 Month Prospective Study. *British Journal of Ophthalmology*.

[3] Kim T. I., Alió Del Barrio J. L., Wilkins M., Cochener B., Ang M. (2019). Refractive Surgery. *Lancet*.

[4] Kamiya K., Shimizu K., Igarashi A., Kobashi H. (2014). Visual and Refractive Outcomes of Femtosecond Lenticule Extraction and Small-Incision Lenticule Extraction for Myopia. *American Journal of Ophthalmology*.

[5] Pedersen I. B., Ivarsen A., Hjortdal J. (2015). Three-Year Results of Small Incision Lenticule Extraction for High Myopia: Refractive Outcomes and Aberrations. *Journal of Refractive Surgery*.

[6] Ang M., Mehta J. S., Chan C., Htoon H. M., Koh J. C., Tan D. T. (2014). Refractive Lenticule Extraction: Transition and Comparison of 3 Surgical Techniques. *Journal of Cataract & Refractive Surgery*.

[7] Kobashi H., Kamiya K., Igarashi A., Takahashi M., Shimizu K. (2018). Two-Years Results of Small-Incision Lenticule Extraction and wavefront-Guided Laser in Situ Keratomileusis for Myopia. *Acta Ophthalmologica*.

[8] Ivarsen A., Asp S., Hjortdal J. (2014). Safety and Complications of More Than 1500 Small-Incision Lenticule Extraction Procedures. *Ophthalmology*.

[9] Vestergaard A., Ivarsen A. R., Asp S., Hjortdal J. Ø. (2012). Small-Incision Lenticule Extraction for Moderate to High Myopia: Predictability, Safety, and Patient Satisfaction. *Journal of Cataract & Refractive Surgery*.

[10] Zhou X. T., Dong Z. X., Yao P. J., Huang J., Xu Y., Xu H. P. (2011). The Clinical Study of Femtosecond Lenticule Extraction for Myopia. *Zhonghua Yan Ke Za Zhi*.

[11] Miao H., He L., Shen Y., Li M., Yu Y., Zhou X. (2014). Optical Quality and Intraocular Scattering After Femtosecond Laser Small Incision Lenticule Extraction. *Journal of Refractive Surgery*.

[12] Han T., Xu Y., Han X. (2019). Three-Year Outcomes of Small Incision Lenticule Extraction (SMILE) and Femtosecond Laser-Assisted Laser in Situ Keratomileusis (FS-LASIK) for Myopia and Myopic Astigmatism. *British Journal of Ophthalmology*.

[13] Li M., Li M., Chen Y. (2019). Five-Year Results of Small Incision Lenticule Extraction (SMILE) and Femtosecond Laser LASIK (FS-LASIK) for Myopia. *Acta Ophthalmologica*.

[14] Xia F., Shen Y., Han T., Zhao J., Xu H., Zhou X. (2020). Small Incision Lenticule Extraction (SMILE) for Moderate and High Myopia: Seven-Year Outcomes of Refraction, Corneal Tomography, and Wavefront Aberrations. *Journal of Ophthalmology*.

[15] Xia F., Chen Z., Miao H. (2024). Ten-Year Outcomes Following Small Incision Lenticule Extraction For Up to −10 Dioptres Myopia. *Clinical and Experimental Optometry*.

[16] Blum M., Lauer A. S., Kunert K. S., Sekundo W. (2019). 10-Year Results of Small Incision Lenticule Extraction. *Journal of Refractive Surgery*.

[17] Papa-Vettorazzi M. R., Güell-Villanueva J. L., Cruz-Rodriguez J. B., Moura-Coelho N., Artells-de Jorge N., Elies-Amat D. (2022). Long-Term Efficacy and Safety Profiles Following Small Incision Lenticule Extraction in Eyes with ≥ 5-Year Follow-Up. *European Journal of Ophthalmology*.

[18] Yildirim Y., Çakmak S., Sucu M. E. (2021). Comparative Study of Small-Incision Lenticule Extraction and Phakic Intraocular Lens Implantation for the Correction of High Myopia: 6-Year Results. *Journal of Cataract & Refractive Surgery*.

[19] Blum M., Täubig K., Gruhn C., Sekundo W., Kunert K. S. (2016). Five-Year Results of Small Incision Lenticule Extraction (Relex SMILE). *British Journal of Ophthalmology*.

[20] Damgaard I. B., Sejersen H., Ivarsen A., Hjortdal J. (2021). 7-Year Results of SMILE for High Myopia: Visual and Refractive Outcomes and Aberrations. *Journal of Refractive Surgery*.

[21] Liu J., Wang Y. (2020). Influence of Preoperative Keratometry on Refractive Outcomes for Myopia Correction With Small Incision Lenticule Extraction. *Journal of Refractive Surgery*.

[22] Blum M., Kunert K. S., Schulze M., Sekundo W. (2019). 10-Year Results of Flex Refractive Surgery. *Journal of Refractive Surgery*.

[23] Shalchi Z., O’Brart D. P., McDonald R. J., Patel P., Archer T. J., Marshall J. (2015). Eighteen-Year Follow-Up of Excimer Laser Photorefractive Keratectomy. *Journal of Cataract & Refractive Surgery*.

[24] Alió J. L., Muftuoglu O., Ortiz D. (2008). Ten-Year Follow-Up of Laser in Situ Keratomileusis for Myopia of Up to −10 Diopters. *American Journal of Ophthalmology*.

[25] Zalentein W. N., Tervo T. M., Holopainen J. M. (2009). Seven-Year Follow-Up of LASIK for Myopia. *Journal of Refractive Surgery*.

[26] Sekundo W., Bönicke K., Mattausch P., Wiegand W. (2003). Six-Year Follow-Up of Laser in Situ Keratomileusis for Moderate and Extreme Myopia Using a First-Generation Excimer Laser and Microkeratome. *Journal of Cataract & Refractive Surgery*.

[27] Zhao Y., Jian W., Chen Y., Knorz M. C., Zhou X. (2017). Three-Year Stability of Posterior Corneal Elevation After Small Incision Lenticule Extraction (SMILE) for Moderate and High Myopia. *Journal of Refractive Surgery*.

[28] Chen Z., Zhao Y., Zhou X., Xia F., Zhao J., Zhou X. (2021). Seven-Year Observation of Posterior Corneal Elevation After Small-Incision Lenticule Extraction in Patients With Moderate and High Myopia. *Journal of Cataract & Refractive Surgery*.

[29] Dupps W. J., Roberts C. (2001). Effect of Acute Biomechanical Changes on Corneal Curvature After Photokeratectomy. *Journal of Refractive Surgery*.

[30] Dupps W. J., Wilson S. E. (2006). Biomechanics and Wound Healing in the Cornea. *Experimental Eye Research*.

[31] Tülü A. B., Çankaya K. İ., Ağca A. (2020). Five-Year Outcomes of Small-Incision Lenticule Extraction vs Femtosecond Laser-Assisted Laser in Situ Keratomileusis: A Contralateral Eye Study. *Journal of Cataract & Refractive Surgery*.

[32] Lee S. B., Hwang B. S., Lee J. (2010). Effects of Decentration of Photorefractive Keratectomy on the Induction of Higher Order Wavefront Aberrations. *Journal of Refractive Surgery*.

[33] Fang L., Wang Y., He X. (2013). Theoretical Analysis of Wavefront Aberration Caused by Treatment Decentration and Transition Zone After Custom Myopic Laser Refractive Surgery. *Journal of Cataract & Refractive Surgery*.

[34] Hu L., Wang Q., Yu P. (2013). The Influence of Intraocular Pressure on Wavefront Aberrations in Patients Undergoing Laser-Assisted in Situ Keratomileusis. *Investigative Ophthalmology & Visual Science*.

